# Supplementary material for: Examining workweek variations in computer usage patterns: An application of ergonomic monitoring software
Source: PLoS One. 2023 Jul 6;18(7):e0287976. doi: 10.1371/journal.pone.0287976 (PMC10325041; doi:10.1371/journal.pone.0287976)
Supplement: Attachment — Submitted filename: Response to Reviewers.docx [file pone.0287976.s001.docx]

DIVISION OF RESEARCH 750 Agronomy Road, Suite 2701 1186 TAMU College Station, TX 77843-1186 Tel. 979.458.1467 Fax. 979.862.3176 http://rcb.tamu.edu

EXEMPTION DETERMINATION

(Common Rule –Effective January, 2018) August 14, 2019

Dear Mark Benden: The HRPP determined on

08/14/2019 that this research meets the criteria for Exemption in accordance with 45 CFR 46.104. This determination applies only to the activities described in this IRB submission and does not apply should any changes be made. If changes are made you must immediately contact the IRB. You may be required to submit a new request to the IRB. Your exemption is good for three (3) years from the Approval Start Date. Thirty days prior to that time, you will be sent an Administrative Check-In Notice to provide an update on the status of your study.

Type of Review: Submission Response for Initial Review Submission Form Title: Evaluation of Computer Usage of Displaced Employees After Hurricane Harvey Investigator: Mark Benden

IRB ID: IRB2018-1623M Reference Number: 085470 Funding: Internal Documents Reviewed: IRB Application (Human Research) - (Version 1.3) TAMU IRB - Review Response Form - IRB - (Version 5.0)

Data Use Agreement - (Version 1.1)

Review Category Category 4: Secondary research for which consent is not required: Secondary research uses of identifiable private information or identifiable biospecimens, if at least one of the following criteria is met: i. The identifiable private information or identifiable biospecimens are publicly available or ii. Information, which may include information about biospecimens, is recorded by the investigator in such a manner that the identity of the human subjects cannot readily be ascertained directly or through identifiers linked to the subjects, the investigator does not contact the subjects, and the investigator will not re-identify subjects If you have any questions, please contact the IRB Administrative Office at 1-979-458-4067, toll free at 1-855-795-8636. Sincerely, IRB Administration
